# Neurostructural changes in schizophrenia and treatment-resistance: a narrative review

**DOI:** 10.1093/psyrad/kkae015

**Published:** 2024-09-06

**Authors:** Tanya Paul, Jia Whei See, Vetrivel Vijayakumar, Temiloluwa Njideaka-Kevin, Hanyou Loh, Vivian Jia Qi Lee, Bekir Nihat Dogrul

**Affiliations:** Department of Medicine, Avalon University School of Medicine, World Trade Center, Willemstad, Curaçao; General Medicine, Universitas Sriwijaya, Palembang City 30114, Indonesia; Department of Psychiatry, United Health Services Hospitals, Johnson City, New York 13790, USA; Department of Medicine, Avalon University School of Medicine, World Trade Center, Willemstad, Curaçao; Department of Medicine, Avalon University School of Medicine, World Trade Center, Willemstad, Curaçao; Department of Medicine, Avalon University School of Medicine, World Trade Center, Willemstad, Curaçao; Department of Psychiatry, University of Rochester Medical Center, Rochester, New York 14642, USA

**Keywords:** schizophrenia, magnetic resonance imaging, PET, SPECT, gray matter, treatment-resistant schizophrenia

## Abstract

Schizophrenia is a complex disorder characterized by multiple neurochemical abnormalities and structural changes in the brain. These abnormalities may begin before recognizable clinical symptoms appear and continue as a dynamic process throughout the illness. Recent advances in imaging techniques have significantly enriched our comprehension of these structural alterations, particularly focusing on gray and white matter irregularities and prefrontal, temporal, and cingulate cortex alterations. Some of the changes suggest treatment resistance to antipsychotic medications, while treatment nonadherence and relapses may further exacerbate structural abnormalities. This narrative review aims to discuss the literature about alterations and deficits within the brain, which could improve the understanding of schizophrenia and how to interpret neurostructural changes.

## Introduction

Schizophrenia, a multifaceted chronic mental disorder with diverse presentations, arises from a complex interplay of risk factors and typically manifests through hallucinations, delusions, disorganization, apathy, withdrawal, and cognitive impairment, affecting >24 million individuals worldwide (Picchioni and Murray, 2007; WHO, 2024). Despite being regarded as a distinct illness for over a century, its parameters and classifications have evolved during this era, and its origin and exact pathophysiology are still unknown (Tandon *et al*., 2013). It is considered a spectrum of disorders rather than one condition (Gillespie *et al*., 2017). The etiology is multifactorial, and genetic predisposition and environmental risk factors significantly affect the disease's development, eventually leading to a wide range of differences in brain structure (Mueser and McGurk, 2004). Dopamine transmission plays a vital role in the pathophysiology of schizophrenia, alongside other neurotransmitter systems, including serotonin and glutamate. Neurostructural changes significantly affecting the gray matter of the frontal and temporal lobes, leading to dysfunctions in circuits (Mueser and McGurk, 2004). Furthermore, some data indicate microcircuit-level dysfunction in the brain involved in schizophrenia (Dienel *et al*., 2022).

There have been more studies in the last decades about how the brain is structurally affected by schizophrenia, especially with the advances in neuroimaging findings, such as in magnetic resonance imaging (MRI). Additionally, our comprehension of the pathophysiology of schizophrenia has progressively expanded over time. Recent studies have also suggested certain findings in brain structure associated with schizophrenia, not only related to the disease itself but possibly also related to antipsychotic medications or relapse periods. Furthermore, not only the efficacy of medications but also medication nonadherence is a significant challenge. The purpose of this review is to provide a narrative discussion of possible neurostructural changes in schizophrenia under various circumstances. We explore brain changes from different perspectives, including the effects of antipsychotics, treatment resistance, and relapse, to understand how these structural changes may be significant for clinical practice. General neurochemical theories are also being discussed to provide a basis for understanding how neurotransmitter systems could be implicated in brain changes. A narrative approach is chosen to provide an overall summary with interpretation. We include some critiques of the cited studies where appropriate, although not extensively. Our search utilized PubMed, Scopus, and Google Scholar using combinations of terms such as “schizophrenia,” “structural changes,” “MRI,” “CT,” “SPECT,” and “PET” to find articles in English from 2000 to 2024. Additionally, we utilized a manual search to find related or cited articles. This review does not aim to cover all relevant literature comprehensively but seeks to provide a broad overview in a nonsystematic manner.

### Neurochemical theories of schizophrenia

Schizophrenia is influenced by a variety of factors, including neurochemical systems, neuroinflammation, and dysfunctions in glial structure. While there are multiple hypotheses regarding the causes of schizophrenia, neurotransmitter theories are the most emphasized. Therefore, we focused exclusively on these theories, acknowledging that no single hypothesis can fully explain all the symptoms or structural changes associated with the disorder. It has been proposed that imbalances in dopamine, serotonin, glutamate, and gamma-aminobutyric acid (GABA) result in the psychiatric manifestations of the disease (Hany *et al*., 2024). Positive symptoms of schizophrenia have been attributed to excessive activation of the dopamine D_2_ receptors via the mesolimbic pathway; on the other hand, negative symptoms have been attributed to low dopamine levels in the mesocortical pathway. Some neuroimaging studies revealed significant dopaminergic dysfunction within nigrostriatal pathways, which is contradictory to the classical mesolimbic dopaminergic hypothesis (Tandon *et al*., 2013). While excessive dopaminergic transmission continues in the striatum, hippocampus, and mesolimbic regions, deficient dopaminergic transmission has been documented in the prefrontal cortex (Nath *et al*., 2021). This is also summarized as subcortical hyperdopaminergia and cortical hypodopaminergia. Interestingly, the primary abnormality in schizophrenia could be related to dopamine synthesis capacity and release rather than dopamine receptors. A meta-analysis has reported presynaptic dopaminergic alterations in the striatum without differences in dopamine transporter availability (Howes *et al*., 2012). Furthermore, there were no D_2_-D_3_ receptor up-regulations in antipsychotic-naïve patients, which may suggest that dopamine receptor abnormalities could be related to antipsychotic treatments rather than the pathophysiology of schizophrenia. These regions are implicated in progressive structural disruptions, as discussed in the next session.

The dopamine hypothesis cannot explain all aspects of schizophrenia, leading to increased interest in the glutamatergic system in recent decades, especially following multiple experimental animal studies. It is important to consider that the glutamatergic hypothesis may be crucial in explaining neurostructural brain changes in schizophrenia (Stone *et al*., 2007). *N*-methyl-D-aspartate (NMDA)-receptor antagonists could be responsible for exacerbating the positive and negative symptoms, suggesting that glutaminergic hypoactivity could contribute to the development of schizophrenia (Patel *et al*., 2014). This hypothesis is also supported by typical actions of NMDA receptor antagonists like ketamine and phencyclidine, which represent findings of positive and negative symptoms of schizophrenia (Tost, Alam, *et al*., 2010). Furthermore, NMDA receptor dysfunction plays a crucial role in cognitive impairment associated with schizophrenia (Javitt, 2023). Also, the clinical presentation of anti-NMDA encephalitis as psychosis is another supporting finding for this hypothesis (Nakazawa and Sapkota, 2020). According to a study by Hu *et al*., antagonists of NMDA receptors can aggravate symptoms in people who already have schizophrenia and induce symptoms such as those of schizophrenia in healthy people (Hu *et al*., 2015). The glutamatergic hypothesis should not be considered separate from the dopaminergic system, as glutamatergic neurons can interact directly with dopaminergic neurons or indirectly via GABAergic interneurons (Kondziella *et al*., 2007). Importantly, NMDA antagonists, including ketamine, have been shown to increase dopamine levels in the striatum, and this change can be blocked by GABAergic interneuron activations (Howes *et al*., 2024). Glutamatergic dysregulation in the hippocampus and prefrontal cortex is reported to cause dopamine release in the striatum, which is a significant finding in connecting the dopaminergic hypothesis (Brisch *et al*., 2014). An extensive meta-analysis indicated that patients with schizophrenia have lower glutamate levels in the medial frontal cortex, higher glutamine levels in the thalamus, and elevated glutamate and glutamine levels in the basal ganglia, suggesting greater variability in glutamatergic metabolites in patients with schizophrenia compared to controls (Merritt *et al*., 2023). A study on carriers of the 22q11.2 deletion, who are at genetic risk for psychosis, suggested that increased glutamate levels in the hippocampus could be related to hippocampal atrophy and disease progression (Mancini *et al*., 2023). A deficiency of D-serine, an NMDA co-agonist, is associated with disrupted hippocampal plasticity and neurochemical abnormalities similar to those observed in schizophrenia (Balu *et al*., 2013). Furthermore, genes involved in glutamate signaling or release, such as dysbindin or neuregulin, have been associated with the pathophysiology (Hany *et al*., 2024). In the last decade, other molecules have been investigated that could be associated with psychosis, such as kynurenine metabolites, which may act on NMDA systems (Dogrul, 2022).

The GABAergic system is also important in schizophrenia, which is mainly studied in post-mortem analyses (Blum and Mann, 2002). In patients with schizophrenia, the expression of glutamic acid decarboxylase 67 (GAD67), which is mostly responsible for GABA synthesis, and GABA transporter-1 (GAT-1), which is primarily responsible for GABA reuptake, in a subset of GABAergic neurons decreased compared to controls, which may indicate an alteration of the inhibitory tone on pyramidal cells (De Jonge *et al*., 2017). A decrease in GABAergic neurotransmission is hypothesized to disinhibit glutamatergic pyramidal neurons, which leads to disruption in cortical activity and dopamine dysregulation (Marques *et al*., 2021). Also, alteration of GAD67 expression is proposed to be associated with cognitive impairment in schizophrenia (Fujihara, 2023). Furthermore, AKT1 (protein kinase B) and GABA_A_ receptor genes are associated with disrupted cognitive functions in a mice model of schizophrenia (Chang *et al*., 2016). A longitudinal study using 7 T MRI found that medicated first-episode psychosis patients had decreased levels of several metabolites, including GABA, in the anterior cingulate cortex (ACC) compared to healthy controls (Wang *et al*., 2023). Another important neurochemical family associated with schizophrenia is neurotrophins. One of the most significant among them is brain-derived neurotrophic factor (BDNF), which may play a critical role in the structural changes associated with the disorder. Reduced BDNF signaling in the cortex could be linked to alterations in the GABAergic system in schizophrenia (Schmidt and Mirnics, 2015). Altered BDNF levels could be associated with disrupted neuroplasticity, impaired synapse regulation, and neurodevelopmental alterations, potentially explaining some neurostructural changes (Nieto *et al*., 2013). Importantly, patients with schizophrenia who have BDNF polymorphisms leading to inefficient BDNF expression show a more significant reduction in frontal gray matter (Ho *et al*., 2007).

Elucidating the mechanisms of action of antipsychotics also improves our understanding of how schizophrenia impacts the brain through different receptors. Especially after data emerged that clozapine interaction on 5-HT_2_ receptors may be associated with the alleviation of negative symptoms of schizophrenia, the serotonin hypothesis became more considered (Wenthur and Lindsley, 2013). One study suggests that the genes linked to schizophrenia are associated with every stage of the serotonin pathway, including serotonin synthesis, transport, and reuptake (Hrovatin *et al*., 2020). A study using brain tissue has also suggested significant variations in 5-HT2_A_ receptor levels in the cortex, alluding to the relevance of serotonin involvement in the pathophysiology of the disease (Dean *et al*., 2016). Serotoninergic overload in the ACC and dorsolateral frontal lobe is suggested to contribute to schizophrenia, and this excess serotonin activity may disrupt the glutamatergic system and lead to neurostructural changes (Eggers, 2013). Not only the serotoninergic but also the cholinergic system is important, as post-mortem studies replicated that there is a generalized decrease of muscarinic acetylcholine receptors in the brain (Scarr *et al*., 2013). Some mechanistic studies suggest specifically that M_1_, M_4_, and M_5_ receptors could be utilized to target the symptoms of schizophrenia (Foster *et al*., 2021). A study investigating blood samples from patients with schizophrenia found antibodies against astrocyte M_1_ and M_2_ muscarinic receptors, which may suggest hypofunction of muscarinic activity (Tani *et al*., 2015). One of the recent promising medications for schizophrenia, xanomeline, is a muscarinic agonist with a high affinity for M_1_ and M_4_ receptors, which may inhibit midbrain dopaminergic circuits and exhibit antipsychotic effects (Paul *et al*., 2024). Furthermore, the alpha-7 nicotinic acetylcholine receptor is also reported to be affected (Higley and Picciotto, 2014). Multiple elements are connected to the pathophysiology of schizophrenia at different levels, including genetic, environmental, hormonal, immune dysfunction, and alterations in neurotransmitters in the brain. This demonstrates that schizophrenia cannot be defined by one specific mechanism. The scientific literature lacks a possible explanation of how disrupted neurochemical systems could affect structural changes in the brain, which remains an important area for future studies.

### Structural changes and neuroimaging findings

Multiple studies in the last decades suggested neurostructural brain changes in schizophrenia. These may include anatomical deficits or their representation of neurochemical changes, but they do not necessarily establish causation, which should always taken cautiously. Studies generally reports cortical tissue deficits, especially in gray matter, and progressive ventricular enlargements (Buckley, 2005; Pantelis, 2005). There are no sole region-specific areas; however, changes in the frontotemporal lobe, ACC, and thalamus are most implicated regions in neuroanatomical studies (Venkatasubramanian, 2010). Thalamus is an important region for schizophrenia, where thalamocortical dynamic disruptions, including oscillatory power and functional connectivity, are reported to be changed (Murray and Anticevic, 2017). In line with this data, evidence suggests that brain oscillation at both low and high frequencies plays a significant role in the pathophysiology (Hirano and Uhlhaas, 2021). A study with diffusion MRI revealed that disrupted thalamo-prefrontal connectivity is a marker of vulnerability to psychotic disorders, indicated by differences between healthy siblings of patients compared to healthy controls without a family history of psychotic disorders (Yao *et al*., 2020). Another study with functional MRI (fMRI), comparing individuals with schizophrenia to healthy volunteers, supported this finding by revealing reduced thalamo-prefrontal connectivity, which was unrelated to local gray matter content within the thalamus or antipsychotic dosage (Woodward *et al*., 2012). However, it is important to note that medications can influence structural changes or connectivity independently of their dose, making studies involving medication-naïve patients crucial. On the other hand, the right dorsolateral prefrontal cortex and cerebellum have disrupted connections, which could be associated with negative symptoms of schizophrenia, and dysfunction of the medial prefrontal cortex could have a role in the pathophysiology (Brady *et al*., 2019; Xu *et al*., 2019). Furthermore, sequencing and microarray studies revealed that transcription of several genes in the prefrontal cortex is abnormal in schizophrenia (Bilecki and Maćkowiak, 2023). This implies that neurostructural changes in schizophrenia cannot be a sole phenomenon but should be understood as part of the broader disease process.

Patients with schizophrenia in their prime years with the onset of the disease showed negative brain development using longitudinal MRI (Kempton *et al*., 2010). Annually, the reduction of brain tissue in patients with schizophrenia is double the rate, which is 0.5%, as opposed to healthy controls at 0.2% (Hulshoff Pol and Kahn, 2007). Moreover, a meta-analysis of 30 longitudinal MRI studies indicated that patients with schizophrenia exhibit a significant reduction in whole brain volume and an enlargement of lateral ventricles compared to controls (Fusar-Poli *et al*., 2013). However, the same study reported that when comparing baseline measurements between individuals with schizophrenia and controls, no differences were observed in gray and white matter volumes, cerebrospinal fluid, or the caudate nucleus. Another meta-analysis corroborated those findings as patients with schizophrenia likely have lower brain volume, specifically whole gray matter and frontal, parietal, and temporal white matter, and expansion in lateral ventricular volume as a function of time (Olabi *et al*., 2011). Those changes over time may suggest a progressive nature of schizophrenia. It should be noted that the decline in brain development is linked to negative symptoms, poorer clinical results, and decreased neuropsychological ability (Cahn *et al*., 2006). Although gray matter reductions are frequently reported, it may be more accurate to refer to them as gray matter abnormalities, as some studies suggest that these changes are not just reductions but also increases in gray matter volume compared to healthy subjects. This is mainly reported for the striatum, including the putamen, right caudate, and globus pallidus (Glahn *et al*., 2008; Hulshoff Pol and Kahn, 2007). However, it should be noted that these studies included patients who were taking antipsychotics, which may affect gray matter volumes. Additionally, a recent study reported increased gray matter concentration in some areas of the temporal, frontal, and parietal cortex, which may reflect a compensatory effect in the early stages of schizophrenia (Li *et al*., 2023). However, this conclusion should be assessed critically due to the cross-sectional methodology, which does not establish causality. A case-control MRI study with 5080 patients with schizophrenia and 6015 healthy controls showed thinner left hemispheric cortices in the rostral ACC and middle temporal gyrus in a small effect size, without association of antipsychotic medications, suggesting left-right asymmetry of the healthy brain is altered in schizophrenia (Schijven *et al*., 2023). This study is critical because it utilized a large sample size, suggesting that prior studies with smaller samples may have reported overestimated asymmetries. However, using cross-sectional datasets limits the ability to comment on causality, highlighting the need for longitudinal studies to elucidate the causality of altered asymmetry. Understanding these structural changes could improve our ability to subcategorize patients within the schizophrenia spectrum or estimate future psychosis risk, providing practical implications for stratifying different treatment approaches in clinical practice.

**Figure 1:**
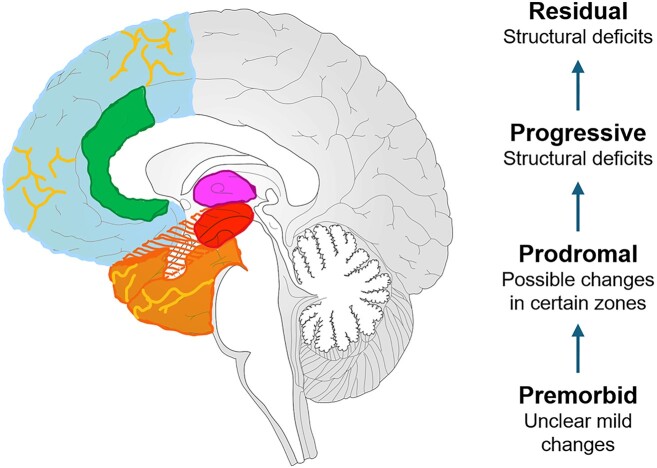
Commonly reported neurostructural deficits in schizophrenia. The premorbid stage could be associated with microstructural changes, although the literature remains inconclusive. The prodromal stage may exhibit early changes in the prefrontal cortex (blue), superior temporal gyrus (hatched orange), putamen (pink), amygdala (red), and hippocampus (red). The progressive and residual stages are associated with dynamic deficits in whole brain volume, most significantly affecting gray matter and gyrification deficits (yellow lines). Along with the illness, frontal (blue), temporal (orange), and anterior cingulate cortices (green) are the most implicated regions related to structural changes in the brain.

Considering neurostructural deficits within the context of the clinical course of schizophrenia could be beneficial, as these deficits unfold gradually from the premorbid to the prodromal phase, where nonspecific behavioral changes emerge and continue into the overt symptomatology of the progressive and residual phases (Lieberman *et al*., 2001). Neurostructural changes in the premorbid and prodromal phases could be critical for diagnostic purposes and early interventions, which makes studies with high-risk populations critical (Fig. 1). A meta-analysis on neuroimaging predictors of high-risk populations with transition to psychosis comparing high-risk populations without transition to psychosis suggested that some neurostructural abnormalities can foresee the development of psychosis in people at high risk (Smieskova *et al*., 2010). It should be noted that the effect sizes were small to medium, and patient populations were mostly heterogeneous at baseline. A cross-sectional MRI study utilizing high-risk populations suggested volumetric deficits in the prefrontal cortex, superior temporal gyrus, amygdala, and hippocampus, although the sample size was small (Keshavan *et al*., 2004). Another cross-sectional MRI study suggested that initial functional abnormalities in the putamen gradually spread over decades, resulting in significant gray and white matter deficits in the insula, cingulate cortex, temporal and frontal lobes, and cerebellum. Although the study was not longitudinal, these observations were made using indirect considerations (Shen *et al*., 2023). On the other hand, a recent large case-control study comparing high-risk individuals with a healthy control group found that neurostructural changes were insufficient for diagnosing or explaining psychosis risk (ENIGMA Clinical High Risk for Psychosis Working Group, 2024). These studies suggest that more data is needed to make clinical considerations, particularly in the premorbid and prodromal phases of schizophrenia, and to specify neurostructural changes in the brain. Additionally, machine learning techniques are promising for analysing and interpreting large datasets, potentially enhancing our understanding of schizophrenia. A recent study using machine learning techniques to classify schizophrenia identified two distinct biotypes based on structural brain changes (Jiang *et al*., 2024). One subgroup is characterized by early cortical loss, beginning in the Broca area and frontoinsular cortex, whereas the other subgroup exhibits early subcortical loss in the hippocampus, amygdala, and other subcortical regions. Interestingly, these results were replicated in antipsychotic-naïve patients, suggesting implicated neurostructural changes most likely associated with the underlying pathophysiology.

The relationship between structural changes and symptomatology in schizophrenia remains unclear. An MRI study comparing patients with schizophrenia to healthy controls found that gray matter deficits varied among different subtypes of schizophrenia. Specifically, disorganization-predominant subtypes exhibited more gray matter deficits in the medial temporal and cerebellar regions, paranoid or hallucinatory subtypes showed additional deficits in the superior temporal cortex, and negative-predominant subtypes demonstrated more deficits in the thalamus (Nenadic *et al*., 2010). However, a recent study could not replicate these findings and found no association between structural changes and disorganization or reality distortion (García-León *et al*., 2024). Furthermore, the initially observed small significance of the negative symptoms and structural correlations did not survive statistical analysis when controlled for cognitive impairment. It is important to note that both studies included patients on first- and second-generation antipsychotics or combinations, making it difficult to exclude medication-related neurostructural alterations. This underlines the importance of neuroimaging studies in patients with first-episode psychosis, which help to exclude medication-induced effects. However, these types of study also have their limitations, as patients are likely to start antipsychotic medications before follow-up imaging, potentially influencing the results.

Nuclear medicine imaging techniques such as positron emission tomography (PET) and single photon emission computed tomography (SPECT) also gave us further data for structural brain changes (Kalyoncu and Gonul, 2021). PET studies suggested lower dopamine synthesis capacity or receptor changes in the hippocampus, prefrontal cortex, and cingulate gyrus (Patel *et al*., 2010). Furthermore, there is an association between schizophrenia and hypofrontality, as evidenced by cerebral metabolic and perfusion deficits, particularly in the frontal cortex (Cumming *et al*., 2021). A SPECT study involving 38 antipsychotic-naïve patients with schizophrenia and 38 healthy controls demonstrated reduced and asymmetric blood flow, particularly in the frontal lobe (Livingston and Scottish Schizophrenia Research Group, 1998). Interestingly, after 6 months of antipsychotic treatment, the study revealed an increase in blood flow in the bilateral putamen among the schizophrenia group, while reduced frontal blood flow persisted. Another study suggested that left temporal lobe hypoperfusion could be a characterizing finding in first-episode schizophrenia and may indicate that functional changes occur before structural changes (Wake *et al*., 2010). It should be noted that this study included patients who were already receiving various antipsychotics with differing durations of treatment, and medication-specific effects on perfusion cannot be excluded, and results should be considered cautiously. However, there are conflicting findings on temporal lobe perfusions in the literature. For example, a single-patient SPECT study suggested that left temporal overactivation could be associated with religious delusions in schizophrenia (Puri *et al*., 2001). However, this finding should be interpreted with caution not only because it is based on a single patient report, but also because the patient was started on depot antipsychotics between the two SPECT imaging. These discrepancies in the literature may be attributed to methodological differences, concurrent medication use, dose-dependent effects, and variations in the duration of medication use. This highlights the need for more standardized methodologies and longitudinal studies with large datasets. In addition to cerebral blood flow, metabolic changes may also play a role in structural differences observed in schizophrenia. Patients with schizophrenia who exhibit predominantly negative symptoms may demonstrate more significant metabolic abnormalities in the brain compared to those with predominantly positive symptoms. A PET study suggested that disrupted emotional expression associated with negative symptoms could be linked to lower glucose metabolism in the ventral prefrontal and orbital cortex (Potkin *et al*., 2002). Additionally, abnormalities in protein expressions and synaptic integrity have also been reported. Specifically, Synaptic Vesicle Protein 2A, which is expressed in synaptic terminals, has been found to be lower in the frontal and temporal cortex in schizophrenia (Howes *et al*., 2023). This finding may suggest alterations in synaptic function and a reduced number of synapses.

There are also ongoing debates that some of the brain changes could be attributed to antipsychotic medications rather than schizophrenia. Since chlorpromazine revolutionized the psychiatry practice, antipsychotics became the mainstay treatment. However, they can have debilitating side effects and could also lead to brain changes in the longer term. Studies suggest that antipsychotics result in rapid structural remodeling of the brain and short-term neural plasticity with acute D_2_ receptor blockade, which can be detected with molecular imaging techniques following a single dose (Handley *et al*., 2013; Tost, Braus, *et al*., 2010). Given that antipsychotic treatment can significantly affect brain structure and function even with acute administration, long-term use of antipsychotics may cause additional neural changes. Some data linked antipsychotic use and brain reductions in bilateral lingual gyrus, thalamus, caudate nucleus, bilateral callosal body, cerebellum, and brain stem, and those reductions could be linked to ventricular enlargement in patients with schizophrenia (Guo *et al*., 2015). A meta-analysis by Huhtaniska *et al*., despite the small, heterogeneous, and underpowered original studies, found that greater antipsychotic exposure was significantly associated with decreased parietal lobe volume and increased basal ganglia volume (Huhtaniska *et al*., 2017). Also, a study with years of computed tomography (CT) imaging suggested that expansion of subarachnoid space correlates with antipsychotic usage (Dabiri *et al*., 2022). Furthermore, some studies suggest that typical and atypical antipsychotics may have different structural effects on the brain. One randomized trial comparing haloperidol and olanzapine in first-episode psychosis found that haloperidol was associated with reduced prefrontal gray matter and whole brain volumes compared to olanzapine, without a dose-dependent correlation (Lieberman, 2005). Despite high attrition rates in the study, sensitivity analysis produced consistent results. Additionally, the duration of untreated psychosis is associated with smaller hippocampal volume, which could result in poorer outcomes with antipsychotic treatment (Yang *et al*., 2021). This suggests that early pharmacological intervention is vital for preventing structural changes and achieving better outcomes. A triple-blind prospective study involving patients with first-episode psychosis and healthy controls found that patients receiving atypical antipsychotics had an increase in pallidum volume, whereas the placebo group showed a decrease in pallidum volume, and healthy controls did not exhibit any changes (Chopra *et al*., 2021). The study also found that an increase in the gray matter volume of the pallidum was associated with a reduction in symptom severity, suggesting focal neuroprotective effects of atypical antipsychotics. However, since the study only used risperidone or paliperidone, which have similar active metabolites, it remains unclear whether these findings are generalizable to all atypical antipsychotics. Additionally, there are mixed results about focal changes with antipsychotics, with some studies showing volume gains and others showing deficits. A prospective trial comparing long-acting risperidone injections to oral risperidone found that white matter volume remained stable in the long-acting injection group while decreasing in the oral risperidone group, which could be attributed to more consistent medication levels (Bartzokis *et al*., 2011). Despite the small study sample, it suggested that not only are antipsychotic medication differences important, but the route of administration can also have a significant effect. This emphasizes the need for standardized methodologies in the literature to interpret the effects of antipsychotics on the brain accurately. Conflicting data about antipsychotic medications and structural deficits critically suggest that advances in neuromodulatory techniques, such as repetitive transcranial magnetic stimulation, could be promising. A sham-controlled randomized trial demonstrated that repetitive transcranial magnetic stimulation is associated with volume increases on the left side, including the hippocampus and parahippocampus, correlating with improvements in negative symptoms (Hasan *et al*., 2017).

It is also critical to acknowledge that structural differences in schizophrenia may not be specific when compared with other psychiatric disorders, such as major depressive disorder or bipolar disorder. This suggests potential common structural similarities between these conditions or more complex higher-order relationships that may involve first- and second-order networks. Studies have reported gray matter volume reductions in the hippocampus, left-sided prefrontal cortex, and medial frontal gyrus in both schizophrenia and depression (Alexandros Lalousis *et al*., 2023). Bipolar disorder also exhibits overlapping gray matter volume reductions with schizophrenia; however, schizophrenia may specifically show reductions in gray matter volume in the left brain, including the amygdala and insula (Yu *et al*., 2010). Interestingly, a study including patients with schizophrenia, bipolar disorder, and matched healthy controls reported reduced gyrification in schizophrenia but not in bipolar disorder or healthy controls (Madre *et al*., 2020). These findings show the need for future research to assess both the distinct and overlapping structural findings of major psychiatric disorders, which could enhance our understanding of schizophrenia.

### Neuroanatomical differences in Treatment-Resistant Schizophrenia

**Figure 2:**
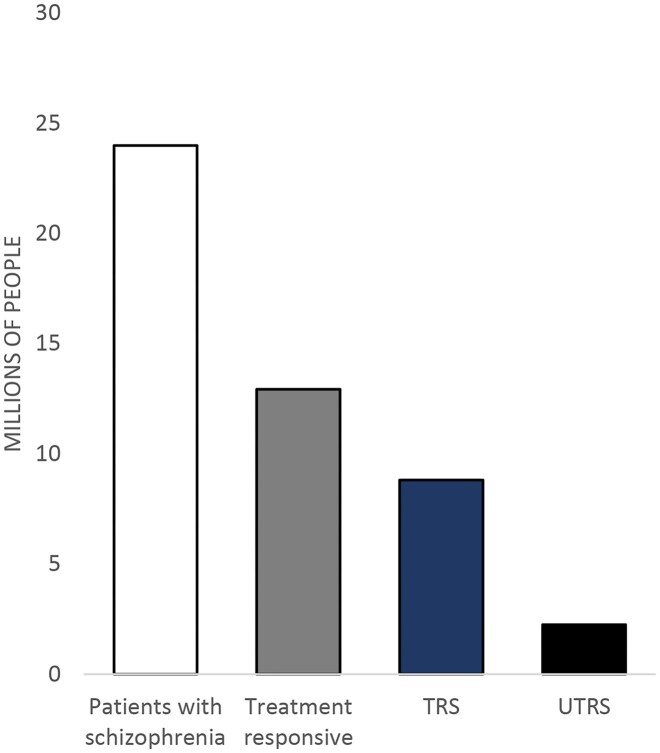
Bar graph of population-based estimates of the prevalence of schizophrenia and TRS. The sum of treatment responsive, TRS, and UTRS equals the patients with schizophrenia (white bar). Reproduced from the data of World Health Organization and Diniz *et al*. (Diniz *et al*., 2023; WHO, 2024).

Treatment-resistant schizophrenia (TRS) is defined as the persistence of symptoms despite two or more trials of antipsychotics for adequate dose and duration without poor compliance or adherence (Potkin *et al*., 2020). A recent meta-analysis study that looked across 50 studies showed that the prevalence of TRS was 36.7% (Fig. 2) (Diniz *et al*., 2023). Clozapine is the only FDA-approved and first-line agent for TRS, alone or combined with other modalities such as electroconvulsive therapy (Nucifora *et al*., 2019). On the other hand, while many TRS cases have a positive response to clozapine, different studies indicate that ~25.6 to 40% of TRS patients do not respond adequately, which is called ultra-resistance (Diniz *et al*., 2023; Siskind *et al*., 2017). Ultra-treatment-resistant schizophrenia (UTRS) is defined as an inadequate response to clozapine with at least 8 weeks of treatment after reaching clozapine plasma levels of a minimum of 350 ng/mL (Campana *et al*., 2021). Patients with UTRS are the most severe population of this disorder and are likely to have multiple prolonged hospitalizations (Chakrabarti, 2021). Differentiating patients based on treatment responsiveness could be beneficial in categorizing structural and neurochemical changes in the brain.

TRS and UTRS could be associated with different changes in the brain. Some studies have suggested that sulcal enlargement, hypogyria, and gyrification deficits in various brain regions are associated with treatment resistance compared to treatment responders (Dazzan, 2014). An MRI study reported reduced gray matter volumes, including cortical areas of frontal, temporal, parietal, and occipital lobes in the TRS and UTRS groups compared to treatment-responsive patients and healthy volunteers (Anderson *et al*., 2015). Gray matter volume loss was most extensive in the superior temporal gyrus, suggesting that the loss of volume in that area could be associated with refractory symptoms and treatment resistance. A longitudinal MRI study reported significantly greater cortical thinning in the left medial frontal and right middle temporal cortex in patients with UTRS compared to TRS, despite no volumetric differences (Ahmed *et al*., 2015). However, it should be noted that UTRS patients were prescribed higher doses of clozapine, which could confound the observed medication-related cortical thinning. It could also be speculated that TRS patients who responded to clozapine might experience attenuated cortical thinning. Therefore, the interpretation of these findings should be approached with caution. Despite this, the available literature suggests a general reduction in frontal lobe volume in UTRS patients compared to clozapine-responsive patients (Pang *et al*., 2023). An MRI study comparing TRS on clozapine with treatment-naïve patients on risperidone suggested that both treatments were associated with decreased white matter volume and increased gray matter volume, where the most significant gray matter volume changes occurred in the occipital regions (Molina *et al*., 2005). The study suggested that more gray matter deficits at baseline result in more improvement in gray matter after treatment with either clozapine or risperidone. Gain in gray matter volumes reported higher in TRS with clozapine treatment, which could be related to more significant gray matter deficits in TRS compared to treatment-naïve patients at baseline. Furthermore, there could be medication-specific effects comparing clozapine to risperidone. A CT study also suggested that prefrontal sulcal prominence is inversely related to clozapine response, suggesting deficits in the prefrontal cortex could be a limiting factor for the effectiveness of clozapine in clinical symptoms (Friedman *et al*., 1991). The authors also suggested that this effect could be explained by the frontal cortex being rich in 5-HT_2_ receptors, and one of the mechanisms of action of clozapine is on 5-HT_2_ receptors. It should be noted that the study compares outcomes after 6 weeks of treatment, a short-term period, raising questions about whether longer-term outcomes might be associated with sulcal prominence. Additionally, other studies propose different structural indicators for treatment resistance or clozapine responsiveness. Even though most studies report deficits in white matter, a study using MRI with 49 patients reported that hypertrophy in the occipital white matter was predictive of treatment resistance, with an area under the curve of 0.848 in the ROC analysis (Molina *et al*., 2008). The same study also concluded that decreases in frontal and occipital gray matter are evident in TRS but not in responsive patients. These discrepancies could suggest that treatment resistance may reflect different biological subtypes of the illness.

Further exploring the changes with clozapine, a SPECT study indicated that patients who responded to clozapine had higher perfusion levels in the thalamus, basal ganglia, and prefrontal areas compared to nonresponders. Also, subcortical and prefrontal perfusions decreased following adequate treatment with clozapine (Rodríguez *et al*., 1997). This study suggested that thalamus and right prefrontal perfusion ratios could be considered predictors of response to clozapine. However, it should be noted that the patients who were defined as treatment-refractory had only trials of typical antipsychotics prior to the categorization. Additionally, intriguing data suggest that treatment resistance may be associated with relatively normal levels of dopamine synthesis capacity, in contrast to the elevated capacity observed in responders (Demjaha *et al*., 2012). Those data have strengthened the hypothesis that TRS could have a different neurobiological basis compared to treatment-responsive schizophrenia (Pandey and Kalita, 2022). Given specific low D_2_ occupancy by clozapine, it has been postulated that D_2_ blockage is insufficient to explain the antipsychotic effects of clozapine, which may be critical to consider the structural changes in TRS (Seeman, 2001).

### Structural consequences of treatment nonadherence

Continued efforts are being put into discovering new antipsychotics and treatment modalities for schizophrenia. However, medication nonadherence is a common issue in patients with schizophrenia and is associated with an increased risk of relapse and multiple admissions (Cahaya *et al*., 2022). Studies suggest that nonadherence rates are estimated at ~50%, although there is considerable variability among studies, which could be related to different definitions of nonadherence, observation timelines, and various methodologies employed across studies (Acosta, 2012). A study that considered major psychiatric disorders and nonadherence rates found that psychotropic medication nonadherence for schizophrenia, bipolar disorder, and major depressive disorder was 56, 44, and 50%, respectively (Semahegn *et al*., 2020). This indicates that medication nonadherence is a general issue, nevertheless schizophrenia has a higher rate of nonadherence compared to other major psychiatric disorders. The Clinical Antipsychotic Trials of Intervention Effectiveness study showed that 74% of patients who were taking antipsychotic treatment discontinued the pharmacotherapy during the first 18 months due to multiple reasons (Lieberman *et al*., 2005). Nonadherence to antipsychotics may be one of the most challenging parts of management, and partial adherence to medications is reported as frequently as complete nonadherence (Higashi *et al*., 2013).

This challenging issue is important because studies show that antipsychotic treatment is less effective with longer relapse times, higher doses are utilized in the second psychotic episode compared to the first episode, and a longer duration of untreated psychosis is associated with worse outcomes (Marshall *et al*., 2005; Wyatt, 1991). Not only TRS or UTRS is significant in the context of structural changes, but also the length of periods of relapse have correlations with brain volume measures, and some studies suggested prolonged hospitalization due to decompensation/relapse is associated with a decrease in gray matter density (Emsley *et al*., 2023; Guo *et al*., 2015). A longitudinal MRI study using retrospective data indicated that prolonged relapse duration, rather than the number of relapses, is linked to decreased total cerebral volume and regional deficits, particularly in the frontal lobe (Andreasen *et al*., 2013). Additionally, the same study demonstrated that higher doses of antipsychotic medications are associated with brain tissue loss, confirming the importance of aiming for the lowest effective dose in schizophrenia management. Even though the causality is not completely clear, alternative explanations could be proposed. These include the possibility that severe subtypes of schizophrenia could experience longer relapses, be less responsive to medications, and consequently have more brain tissue loss. These findings suggest that preventing relapses with a minimal treatment regimen would be the optimal approach for the management, not only to minimize side effects but also to prevent or improve structural deficits. Furthermore, hospitalization rates could also be an important indicator of treatment compliance and success. One study with voxel-based morphometric MRI suggested that a decrease in the gray matter density in the left superior frontal gyrus is associated with increased hospitalizations, which could be considered relapses (Van Haren *et al*., 2007). Additionally, cortical thinning in the left frontotemporoparietal regions is associated with impaired insight in schizophrenia, particularly evident in TRS (Kim *et al*., 2023). Multiple studies have also indicated deficits in the frontopolar cortex and cortical midline structures, including the orbital and medial prefrontal cortex, dorsomedial prefrontal cortex, and anterior and posterior cingulate cortex in schizophrenia (Raij *et al*., 2012). Some of those regions are associated with the model of self or self-referential processing, which is crucial for insight (Northoff and Bermpohl, 2004). These findings may potentially contribute to nonadherence in patients due to a lack of insight. All of the data corroborate structural brain changes in schizophrenia, TRS, UTRS, and relapses.

Comorbidities associated with schizophrenia also affect the complexity of treatment nonadherence. Nonadherence is associated with socioeconomic status, lack of family support, side effects of medications, substance use, poor insight, and cultural stigmatization; however, there are no single effective methods to assess the risk (Li *et al*., 2024; Phan, 2016). Studies report that psychiatric comorbidities such as depression and anxiety are more prevalent among patients with schizophrenia, with depression topping the list compared to the general population (Buckley *et al*., 2009). Furthermore, people with schizophrenia not only suffer from mental and physical complications but also from social and financial struggles, where assessing and analyzing the social barriers is crucial in the management of schizophrenia (Weittenhiller *et al*., 2021). Family support also plays a crucial role in treatment adherence, as one of the studies reports that medication nonadherence rates are lower at 34.7% in patients with schizophrenia who live with family caregivers compared to those who did not at 60% (Li *et al*., 2024). Also, cognitive impairment may have an impact on adherence, and unfortunately, some emerging data suggest antipsychotic medications could worsen cognitive functioning in specific domains (Allott *et al*., 2024; El-Missiry *et al*., 2015; Perkins, 2002). It could be proposed that medication nonadherence could lead to relapses that could further exacerbate structural and neurochemical changes in the brain. A significant factor for medication adherence seems to be having a good therapeutic relationship, and poor adherence seems to be associated with a lack of insight into illness (Higashi *et al*., 2013). A *post hoc* analysis in multiple clinical trials reported that the best predictor for adherence is an improvement in positive symptoms, hostility, and depressive symptoms, regardless of which atypical antipsychotics are utilized (Liu-Seifert *et al*., 2012). In cases of noncompliance, long-acting (depot) antipsychotic injections can be used. However, combining educational and behavioural strategies can also be impactful. These strategies include motivational interviewing, cognitive behavioural therapy, psychoeducation, and daily medication reminders (Correll *et al*., 2021; Loots *et al*., 2021). Assessing risk factors associated with nonadherence to identify potential interventions is important. Conducting an initial assessment of these risk factors could benefit the long-term management of nonadherence, and this approach may help slow neurostructural deterioration. Simplifying medication regimen can also be helpful (Pai and Vella, 2022). Given that some studies suggest antipsychotics can lead to neurostructural changes in the brain, using the minimum necessary dose may not only improve adherence but also help prevent long-term changes in the brain. Monitoring prodromal symptoms of schizophrenia using tools such as the Early Signs Questionnaire could be significant for preventing relapses and may indirectly help prevent neurostructural deterioration (Lamberti, 2001).

### Critics and Future Directions

Numerous studies are reporting varying and sometimes conflicting structural changes in schizophrenia. Additionally, the changes in schizophrenia are dynamic, with both reductions and increases in brain volume potentially occurring throughout the illness. It is important to remember that the clinical significance of focal neurostructural changes and the processes leading to these changes remain largely unknown. Given the mixed findings reported in various studies, it is unclear whether all patients with schizophrenia exhibit the same anatomical findings or if these findings represent different anatomical subtypes. These discrepancies may relate to different subtypes of the schizophrenia spectrum, the severity of the illness or relapse, medication history or current regimens, and comorbid medical and psychiatric conditions. Notably, there are multiple variables in most of the studies, making them hard to interpret in general. It is crucial to consider factors such as patient gender, age, relapse history, severity of schizophrenia, substance use, environmental risk factors, hospitalizations, treatment compliance or partial compliance, outpatient engagement, social support, and even different scanner types or image processing protocols (Table 1). No single study should be used to make conclusions about neurostructural changes; however, the overall interpretations of these studies can provide valuable clinical insights. Those changes could also represent a significant avenue for developing neurostimulation techniques and stratifying personalized treatment plans, making it important to combine neurostimulation studies with neuroimaging. Despite ongoing efforts, diagnosing schizophrenia based on current data for structural alteration remains a significant challenge, as these changes are not specific. Imaging studies on medication naïve patients could usually be appropriate for a better understanding of the brain; however, there are significant ethical reasons to consider. Furthermore, when interpreting studies, it is crucial to be mindful of reverse inference and how it may lead to faulty interpretations (Bernard, 2020). It should also be noted that clinical trials often include selected patient populations. This selection process can involve patients with varying levels of insight, medication compliance, and severity of schizophrenia, which may impact the generalizability of the findings. To make those results clinically valuable, longitudinal studies assessing causality with generalizable samples are needed.

**Table 1:** Possible reasons for inconsistent data about the neurostructural changes.

Demographics (e.g. age, gender, race)
Different biological subtypes
Duration or severity (e.g. first episode psychosis, chronic schizophrenia)
Dynamic nature of structural changes (e.g. volume decreases or increases)
Current or prior antipsychotic usage and doses
Medication specific effects (e.g. first or second-generation antipsychotics)
Route of administration (e.g. oral, long-acting injections)
Treatment response (e.g. TRS, UTRS)
Adherence (e.g. full adherence, partial adherence, nonadherence)
Relapse history (e.g. frequency, duration)
Comorbid medical or psychiatric conditions
Current or prior substance use
Lack of generalizability (e.g. selected populations for clinical trials)
Sample variance (e.g. heterogeneity)
Different effects sizes (e.g. small, large)
Medication changes between follow up imaging
Study designs (e.g. cross-sectional, case control)
Imaging method (e.g. MRI, CT)
Machine type (e.g. brand, machine power, software)
Imaging protocol (e.g. image acquisition, processing)

## Conclusion

Schizophrenia has multifaceted consequences on the brain. Multiple hypotheses can explain different symptoms and presentations of schizophrenia; however, the most robust theory is still the dopamine hypothesis. Numerous studies indicate that both structural and neurochemical changes occur in the brains of individuals with schizophrenia. Recent advances in imaging techniques have broadened our understanding of these progressive differences, particularly in specific brain areas. The changes in schizophrenia seem to include multiple areas of the brain, mostly focused on gray matter volume deficits. Differences are frequently reported in frontal and temporal lobes, involving the cingulate cortex, thalamus, and parahippocampal gyrus. Furthermore, untreated psychosis and prolonged relapses are associated with some changes, including hippocampal volume deficits. Treatment resistance is another entity that can affect the brain differently, especially via gyrification deficits. On the other hand, antipsychotic treatments could improve those changes, and gray matter volumes could increase with adequate treatment. However, some data suggest antipsychotic medications could also contribute to some structural deficits in the brain. All these make treatment nonadherence at the center of the management of schizophrenia, as it is frequent and associated with further changes in the brain. Conflicting or inconsistent data about specific structural deficits may be attributed to inconsistent methodologies or highly heterogeneous datasets, making comparisons challenging. Studies on structural changes could improve our ability to categorize, manage, and follow the progression or treatment response in schizophrenia. A better understanding of those changes could stratify our medication choices based on specific cases.
